# Investigating a Potential Bias in Resurrection Experiments to Measure Adaptive Evolution of Flowering Time

**DOI:** 10.1002/ece3.73101

**Published:** 2026-03-23

**Authors:** Sarah M. Ravoth, Ariane Mooney, Emily J. Austen, Alice DesRoches, A. J. Westerhof, Arthur E. Weis

**Affiliations:** ^1^ Department of Zoology University of British Columbia Vancouver British Columbia Canada; ^2^ Faculty of Biosciences Norwegian University of Life Sciences Ås Norway; ^3^ Biology Department Mount Allison University New Brunswick Canada; ^4^ Department of Ecology & Evolutionary Biology University of Toronto Toronto Ontario Canada; ^5^ Department of Biology University of Western Ontario London Ontario Canada

**Keywords:** adaptive evolution, climate change, missing data, phenology, resurrection experiment, seedbank

## Abstract

Species persistence under climate change often depends on rapid evolutionary responses to increasingly extreme conditions. Resurrection experiments are a valuable approach for tracking the rate of adaptive evolution: ancestral propagules are stored, then later revived and grown alongside descendants, exposing shifts in trait values that indicate evolutionary change. However, even under the best storage conditions, ancestral propagules age and eventually a fraction die. If propagule longevity in storage is non‐random and genetically correlated with a trait of interest, bias is introduced to the ancestral trait baseline, consequently distorting estimates of adaptive evolution. To understand the degree to which non‐random ancestral propagule loss biases resurrection experiments, we simulated long‐term seed aging of the annual *Brassica rapa* , testing whether seed “birth order”, a proxy for maternal resource allocation, influences seed survivorship. We then evaluated whether birth order correlates with flowering time, a trait previously demonstrated to evolve rapidly in response to climate change. Rapidly aged seeds had 26% reduced survivorship and flowered approximately one day later than unaged control seeds. This plastic storage effect disappeared in the F2 generation. Furthermore, simulations testing differential storage survivorship with birth order (i.e., between first and last produced seeds) reveal the bias on flowering time is low on average (< 1 day), even under extreme mortality asymmetries. Together, results from this study system suggest the resurrection approach remains a reliable experimental methodology in global change biology.

## Graphical abstract

Resurrection studies are powerful tools for scientists to track adaptive evolution for species with dormant life stages. However, this method is contingent on long‐term storage of propagules, potentially introducing biases that can distort signals of evolutionary change. We test a mechanistic basis for these biases and their influence in distorting evolutionary signals in flowering phenology.

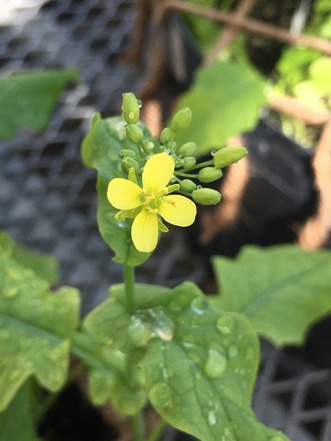

## 1 Introduction

Novel stressors associated with global change, such as pollution, land use change, and climate change, threaten species persistence globally (Sala et al. 2000; Jaureguiberry et al. 2022). Elevated temperatures and altered precipitation patterns are predicted to be the principal drivers of future biodiversity loss, elevating extinction risk (Urban 2015) through population declines (Spooner et al. 2018; Müller et al. 2024) and erosion of genetic diversity (Bellard et al. 2012). Range shifts to more favorable climatic conditions and/or phenotypic plasticity can ameliorate population declines (Parmesan and Yohe 2003; Merilä and Hendry 2014). However, persistence may ultimately depend on rapid evolution of key adaptive traits, particularly for immobile species (Catullo et al. 2019). Thus, documenting the rate of contemporary evolution and the types of traits that contribute most to adaptation provides vital information for predicting species loss and informing conservation management.

### 1.1 Using the Resurrection Approach to Detect Evolution in Natural Populations

The resurrection approach is a direct method of measuring rapid phenotypic evolution that can be applied to species with a dormant life stage. In a resurrection experiment, propagules of an ancestral population are stored and later revived to be grown side‐by‐side with descendant generations (Franks et al. 2018). Because the rearing environment is the same for both ancestors and descendants, phenotypic differences reflect genetic divergence (Franks et al. 2018; Weis 2018). This method can be applied across a broad variety of study systems, from investigating thermal tolerance in marine diatoms (Schaum et al. 2018) to pollutant resistance in *Daphnia* (Piscia et al. 2015).

Plants are particularly apt subjects for the resurrection approach because seeds can remain viable for years to decades under proper storage conditions. Over 50 resurrection studies have focused on plants over the past two decades (Pennington et al. 2025). These have documented evolutionary shifts in glyphosate herbicide resistance in morning glory (Kuester et al. 2016), drought avoidance and escape traits in scarlet monkeyflower (Anstett et al. 2021), and flowering phenology for several species (e.g., Hamann et al. 2018; Dickman et al. 2019; Lambrecht et al. 2020; Vtipil and Sheth 2020; Kooyers et al. 2021). To facilitate such studies, Project Baseline established a seed bank for multiple populations of 65 plant species across the continental United States. Seeds are stored under conditions likely to preserve viability past the mid‐21st century (Etterson et al. 2016). Investigators can withdraw seeds from the collection to use as the ancestral generation in resurrection experiments or to study geographic variation across multiple populations (e.g., Soper Gorden et al. 2016).

However, the resurrection approach is not without caveats. Like all living things, propagules are not immortal. In seeds specifically, biochemical changes degrade mRNA and modify proteins over time, thereby depleting nutrient reserves and decreasing detoxification efficiency within a seed (Rajjou et al. 2008; Yin et al. 2015). Although individual genotype and developmental environment contribute to aging resilience, this process can be slowed under proper storage conditions, including low temperature and low humidity (Gulden et al. 2008; Rajjou et al. 2008; Walters et al. 2010). Nevertheless, some deterioration is possible, reflected by reduced germination rates. Storage‐induced changes can bias estimates of the ancestral generation's mean phenotype—the baseline for comparison—in two ways.

First, plants may plastically modify development and post‐emergent phenotypes in response to stress induced by long‐term storage. This “storage condition” bias can be ameliorated by raising a “refresher” (F1) generation of both ancestors and descendants to maturity under the same environmental conditions. This equalizes the quality of the resulting seed, which is then reared in the “test” (F2) generation, where the phenotypes are measured (Franks et al. 2018; Weis 2018).

The second bias in estimating the ancestral mean phenotype can result from genetically non‐random survival during storage (Walters et al. 2010), leading to an “invisible fraction” problem. The invisible fraction describes the portion of a population that cannot be measured, for instance, due to early mortality (Grafen 1988). If seed mortality in storage is random with respect to genotype, the surviving subsample will be an unbiased representation of the ancestral population. However, unequal loss of certain genotypes or cultivars after storage has been documented in some crop species (e.g., flax (Balouchi et al. 2017), rice (Lee et al. 2019), barley (Nagel et al. 2015)). This poses a problem to resurrection studies: in a wild population, if seeds vary in some trait conferring seed longevity, long‐term storage will act as a selective sieve, favoring individuals possessing certain trait values. Should the loci affecting longevity in storage also be associated with the post‐emergence phenotypes of interest, the baseline for estimating evolutionary change in that population—the core objective of a resurrection experiment—will be distorted. The severity of bias increases with the storage mortality rate and the strength of genetic correlation between the survival and post‐emergence traits (Weis 2018).

To our knowledge, only one empirical study has investigated an invisible fraction bias arising from non‐random seed mortality during storage. Franks et al. (2019) applied a high humidity and temperature treatment to rapidly induce physiological aging (Rajjou et al. 2008; Yin et al. 2015) in rapid‐cycling *Brassica rapa*. The aging treatment imposed strong selective pressure—31% of aged seeds germinated and only 14% survived to seed set, relative to the controls, which germinated and survived to seed set at rates of 93% and 87%, respectively. Plants growing from aged seed (i.e., F1 refresher generation) flowered significantly later (2.9 days) than controls. The difference persisted into the F2 test generation, although diminished (0.6 days). The persisting difference in the F2 test generation confirms that simulated storage passed seeds through a selective sieve. The larger difference in the F1 refresher generation indicates an additional storage effect delaying flowering time.

### 1.2 How Could an Invisible Fraction Bias Arise?

Here, we propose and test a mechanism that could generate a genetic correlation between seed survival and flowering time, a potential source for an invisible fraction bias. Bias could arise through the priority effect (Austen et al. 2015), whereby seed quality depends upon “birth order”. Plants typically deploy flowers over a period of days to weeks (e.g., Weis et al. 2014). Seeds initiated within the first opened flower on a plant have full access to maternal resources during development. In contrast, seeds initiated within the last flower must compete with their earlier‐produced siblings for maternal resources. Thus, last‐produced seeds on a given maternal plant may accumulate fewer resources than first‐produced seeds. When seeds are placed in storage, and their resource reserves degrade with time, these provisioning differences could induce a negative relationship between provisioning priority and seed longevity.

Reduced provisioning of last‐produced seeds can lead to a genetic correlation between seed survival and flowering time of the germinant. The correlation arises because, as a flowering season progresses, the population average genetic value for flowering time in the pollen pool shifts from early to late (Ison and Weis 2017). The first flower on the earliest flowering maternal plant can be pollinated only by sires that likewise flowered early, whereas seeds produced by the last flowers on that same maternal plant may be sired by a pollen donor with a later flowering‐time genotype. Similarly, the last flower on late‐flowering plants is more likely to be pollinated by a later‐flowering pollen donor than are early flowers on that same plant. Simply put, the flowering time alleles inherited from the pollen parent depend on birth order within a maternal plant—first‐born have genetically earlier fathers than last‐born, on average. As a result, well‐resourced, first‐produced seeds, which may survive longer in storage due to provisioning priority, tend to also carry paternally inherited loci for earlier flowering. In contrast, the comparatively poorly resourced, last produced seeds tend to have paternally inherited loci for later flowering and lower chance of survival. Consequently, seed longevity and flowering time would become genetically correlated.

### 1.3 Testing the Potential Contribution of Priority Effects to an Invisible Fraction Bias

**FIGURE 1:**
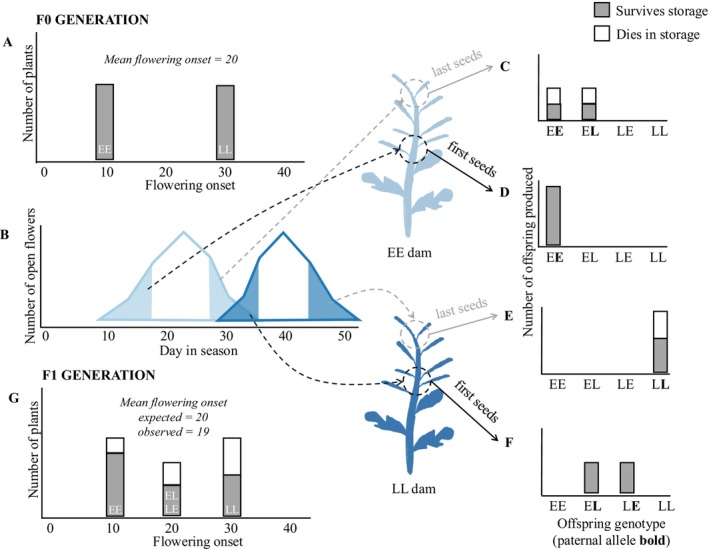
Theoretical mechanism for the occurrence of an unmeasured “invisible fraction” for flowering time in the F1 generation. (A) The population is initiated with an equal number of early‐flowering plants (genotype EE, flowering onset = 10 days) and late‐flowering plants (genotype LL, flowering onset = 30 days). Plants of both genotypes produce flowers for 25 days following their flowering onset and the peak flower deployment of each plant falls in the middle of its flowering period. (B) The density plot depicts the availability of flowers from the EE and LL genotypes in the population over the course of the flowering season. Because plants can only exchange pollen when they have open flowers on the same day, seeds within the first‐produced flowers on EE plants will be sired only by other EE plants, while seeds within the last flower on EE plants may be sired by EE or LL plants. On LL plants, seeds within first flowers are sired by EE or LL, and seeds within last flowers are sired only by LL. (C–F): Distributions of expected genotypes for four “batches” of seeds, respectively: Last produced seeds on EE dams, first produced seeds on EE dams, last produced seeds on LL dams, and first produced seeds on LL dams. On both dam genotypes, last produced seeds have a reduced probability of survival after storage; the paternally‐inherited L allele is disproportionately lost during storage. (G) The expected (based on seeds produced) and observed (based on seeds surviving storage) distribution of genotypes and flowering onset in the F1 generation. Owing to reduced survival of last‐produced seeds on dams, the observed mean flowering time is one day earlier than the expected in this parameterization. For simplicity, this model was built assuming a simple, single‐locus inheritance with incomplete dominance for flowering time, but the same principles are expected to apply in more realistic settings in which flowering time is inherited as a quantitative trait.

Figure 1 presents a hypothetical example for a population comprising two flowering time genotypes, early (EE) and late (LL), with flowering time phenotypes of 10 and 30 days, respectively. If all seeds survive storage, and assuming flowering time is determined by a single locus with partial dominance (EL flowering time of 20 days), the mean flowering time of the F1 generation would be 20 days. Suppose first produced flowers produce high‐quality seed that survives storage and, due to assortative mating, have a high proportion of early flowering alleles (EE or EL genotype). In contrast, last produced flowers produce poorly provisioned seed with more L alleles (EL or LL genotype). If only 50% of seeds from last produced flowers germinate after storage, the F1 mean will be 19.2 days—although the genetic value for flowering time was 20 days for the seeds that went into storage, those surviving storage flower 0.8 days earlier than the true average. Taking this toy example to a greater extreme, if all the last produced seed dies in storage, the mean for the F1 generation falls to 17.5 days, an invisible fraction bias of 2.5 days.

Using a three‐generation experiment, we evaluate the threat of the invisible fraction problem to resurrection experiments by asking two questions: (1) Do seeds from last‐deployed flowers show lower survivorship under a simulated aging treatment—i.e., does “birth order” influence seed survival?; and (2) Do the offspring grown from a maternal plants' first produced seeds flower earlier than the those from the last‐produced seeds—i.e., is there a correlation between seed “birth order” and the genetic contribution from the pollen parent?

## 2 Materials and Methods

This project was conducted using *Brassica rapa* , commonly known as field mustard. *B. rapa* is a perfect‐flowered obligate outcrosser naturalized across North America. Seeds produced on a plant share the same maternal genes, but differ in genes received from the paternal plant (pollen‐donor). In wild populations, *B. rapa* typically produce their first flower approximately 30 days after emergence, continuing to produce flowers long after setting the first fruits (Bonner et al. 2019). We define flowering time as the number of days between germination and first anthesis.

To maximize the chances of detecting even a weak invisible fraction bias, this multigeneration experiment used two genetic lines, selected for early and late flowering time (Austen and Weis 2015). The early line transitions from vegetative growth to flowering approximately 14 days ahead of the late line, and both continue to produce flowers for approximately 30 days (Bonner et al. 2019), although the late flowering line can extend its flowering period longer under favorable conditions. While we refer to these as the EE and LL, we do not imply that they differ by a fixed difference at a single locus. Simply, it lets us designate potential hybrids between the lines as EL.

### 2.1 Experimental Overview

Our experiment was modeled on recommended protocols for resurrection experiments, extending across three generations. The F0 generation represents an ancestral wild population from which seeds are collected for storage, although here we source F0 individuals from selected lines to parse an invisible fraction bias. We recorded the flowering time of F0 plants and collected their seeds by provisioning priority group, i.e., from the flowers deployed during the first and last intervals of their flowering period, information not typically available from long‐term seed collections. A subset of these seeds was then subjected to a rapid aging treatment designed to accelerate the physiological deterioration seen under long‐term storage. Artificial aging protocols that expose seeds to hot, humid conditions for short periods well simulate the process of natural aging in the seedbank, because the same physiochemical changes (e.g., mRNA oxidation, protein modifications, sugar hydrolysis) occur in both processes (Delouche and Baskin 1973; Rajjou et al. 2008; Yin et al. 2015; Franks et al. 2019). Rapidly aged and control seeds were germinated, and their survival rates recorded. These germinates gave rise to the F1 refresher generation. Any differences in plant phenotype between the experimental and control treatments in this generation include storage effects induced by deterioration under storage. Once this refresher generation matured, we planted their seeds without any further manipulations to produce the F2 test generation free from plastic storage effects.

We used a balanced experimental design to test for differences in flowering time phenotypes based on provisioning priority (first vs. last produced) and aging treatment (aged vs. control). The balanced design increased the power to detect effects of these predictors on flowering time, which was a major objective for this study. However, equal sample sizes obscure potential invisible fraction effects. We therefore expect that any observed flowering time difference between aged and control treatments in the F1 would be exclusively due to plastic responses to the aging treatment, and that there should be no difference in mean flowering time between aged and control treatments in the F2 generation (in which plastic responses have been alleviated). In contrast, in typical resurrection experiments, the investigator will not know if last‐produced seed, which tend to carry paternally‐inherited L alleles, are underrepresented among the successful germinants. They would not know if data were missing and so could not detect an invisible fraction effect. The balanced design in our study assured equal representation and thus delivered robust estimates of group mean phenotypes. However, it also necessitated additional steps to estimate any invisible fraction effect.

To evaluate the potential effect of unequal survival through storage on estimates of the “ancestral mean phenotype” (i.e., aged treatment) we performed two simulations. The first resampled flowering day data from the F2 generation, drawing from the two priority groups in proportion to the survival rates of the F1. Differences between the aged and control treatments would thus reflect underrepresentation of the latter priority group and thereby reveal any invisible fraction bias in estimates of mean phenotype. The second simulation explored the bias under a spectrum of survival differences (i.e., proportion of data sampled) between priority groups.

The following sections detail methods for each of the three generations, statistical analysis, and simulation design.

### 2.2 F0 Generation

For the parental generation, we raised 32 each of early‐ and late‐flowering plants (EE and LL) in the greenhouse under a 15‐h photoperiod at 22°C–26°C. Plants were planted in two round containers (75 cm radius), or blocks, with 16 EE and 16 LL plants positioned randomly within each block. Plants were grown together rather than individually to induce asymmetries in resource competition (e.g., soil nutrients, space) and thus enhance potential priority effects. Seeds were planted on experimental day 0 in block 1 and day 14 in block 2. Each plant received approximately 0.12 g of 20:20:20 water‐soluble Miracle‐Gro (6 g/L) fertilizer on experimental days 7 and 21. Once the first flowers opened, we randomly pollinated plants daily within each block using a craft feather.

We recorded germination day, the day of the first flower, and the number of seeds and seed pods produced. We also marked the newest flower and counted the number of open, receptive flowers on each plant every five days. This allowed us to estimate the ratio of early and late flowering alleles in the pollen pool through time, and consequently, the ratio of offspring phenotypes. After 90% of plants had finished flowering, we stopped watering and allowed seeds to mature and plants to completely dry out. Seeds were collected with respect to maternal plant ID and birth order.

### 2.3 F1 Refresher Generation

For each aging treatment × priority group × maternal line × block grouping, we created an F1 generation of 44 plants (704 plants total in the F1 generation). These 44 individuals were drawn from 5 to 7 F0 maternal plants (2–15 offspring per maternal plant) that met certain criteria for inclusion described below. To ensure the target of 44 plants per F1 population was met, we planted several seeds (“replicates”) per required plant (3 per plant required in the control treatment and 5 per plant required in the aging treatment), and later thinned to one germinant per required plant.

**FIGURE 2:**
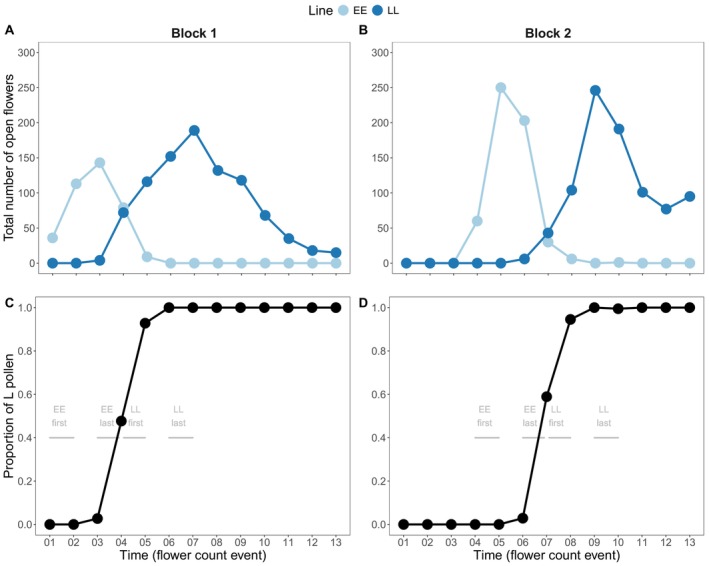
Total number of open flowers and proportion of late‐flowering pollen produced per flowering line and block in the F0 generation. Panels A and C show plants from block 1, and B and D show plants from block 2. Panels A and B show the total number of open flowers per flowering line and block. Panels C and D show the proportion of pollen produced by the late flowering line (L), estimated by the proportion of open flowers of EE versus LL plants. Flower counts occurred every five days. Gray lines indicate when F1 individuals were harvested relative to the proportion of L pollen, and labels indicate the maternal line and seed priority.

The criteria for inclusion of maternal plants were as follows. Early flowering maternal plants must: (i) have set their first seeds before late plants began flowering (before count event 2 for block 1, or before count 5 for block 2; Figure 2), (ii) produced their last seeds when both early and late plants were flowering (between count events 3 and 4 for block 1, and between counts 6 and 7 for block 2; Figure 2), and (iii) produced at least 16 seeds per priority group to allow contribution of a few replicates per treatment. Late flowering maternal plants must: (i) have set first seeds while both early and late flowering plants were flowering (i.e., between counts 4 and 5 in block 1, and between counts 7 and 8 in block 2; Figure 2), (ii) produced their last seed set when only late flowering plants were in flower (i.e., after count 6 in block 1, and after count 9 in block 2), and (iii) produced at least 16 seeds per priority group. Note that overlap in flowering schedules between EE and LL plants was limited (Figure 2A,B; see Discussion). Furthermore, we ensured equal temporal spacing between the collection of first‐ and last‐produced seed for both EE and LL lines, assuming that decay rates in maternal resources over time are consistent between genetic lines.

To simulate deterioration under long‐term storage, we used a similar protocol as Franks et al. 2018. The aged seeds (*n* = 2494) were kept at 40°C for 160 h at 100% humidity, then stored in the fridge at approximately 4°C for several days before planting. Control (unaged, *n* = 1132) seeds were stored at 4°C throughout.

On experimental day 0, we planted 3–5 replicate seeds per required F1 plant in soil in flat 30 × 60cm trays according to maternal plant ID, block, aging treatment, and priority group. We watered and recorded germination daily for two weeks (day 14). On day 14, we randomly selected one replicate per required plant and transplanted it into an individual Ray Leach “Super Cell” UV pot (Stuewe & Sons, OR, USA, hereafter “conetainer”). Conetainer rack number, hereafter position in the greenhouse, was randomized.

The F1s were grown in a greenhouse at 22°C–26°C with a 15‐h photoperiod. Each plant received approximately 0.12 g of 20:20:20 water‐soluble Miracle‐Gro (6 g/L) on experimental day 12. Plants were watered and checked for flowering initiation daily. If the first flowering day was missed (i.e., first flowering day was not recorded but the plant had obviously been flowering for more than one day), we estimated the date using accumulated knowledge of *B. rapa* flowering schedules (Weis and Kossler 2004). Briefly, plants with two open flowers were assigned to two days prior, 3–4 open flowers three days prior, 5–6 flowers four days prior, and 7–8 flowers five days prior. Plants that did not flower by the end of the experiment were considered right censored.

Once plants started flowering, we used a craft feather to randomly pollinate plants daily according to aging treatment and priority group. When 90% of the plants had finished flowering, we stopped watering and allowed the seeds to mature and the plants to dry completely. We counted total seed pods produced by each plant and collected seeds in packets based on plant ID.

### 2.4 F2 Test Generation

The purpose of the F2 generation was to confirm that any plastic effects of the aging treatment on flowering time were restricted to a single generation and generate flowering time data free of this plastic storage response to measure an invisible fraction via simulation (see *Data analysis* below). In this generation, we randomized seeds planted with respect to maternal ID (i.e., the F1 generation plant ID) so that each maternal plant had equal seed contribution to the F2 generation without complicating the analysis by including a random effect of maternal ID. Five seeds were selected from each parental plant and pooled into four aging treatment and priority groups (i.e., aged–first priority, aged–last priority, control–first priority, control–last priority). From this pool, we randomly drew seeds to plant 245 conetainers per priority group × aging treatment (980 conetainers total). We planted two seeds per conetainer, monitored germination for eight days, and thinned to one seedling 10 days after planting. Conetainer position in the greenhouse was randomized. We watered conetainers daily. On day 15, each plant received 0.12 g of 15:15:30 water‐soluble Miracle‐Gro (6 g/L) fertilizer. We recorded the day each plant began flowering. We estimated the fflowering day for plants where the first flowering day was missed using the same scheme as for F1s. Plants that did not flower by the end of the experiment were considered right censored. We terminated watering when 90% of the plants had finished flowering.

### 2.5 Data Analysis

We tested for inadvertent fitness differences (i.e., seed pod production) that may have arisen between blocks in the F0 generation using a linear model and checked assumptions of residual heteroscedasticity and normality. To determine differences in germination success between aged and control groups in the F1 generation, we used a generalized linear mixed effects model with a binomial distribution and logit link (Bates et al. 2015). We assessed assumptions regarding appropriate link function (met), appropriate estimation of variance (met), and random effects approximating a normal distribution (not met).

We used non‐parametric Kaplan–Meier survival analysis to investigate differences in flowering time between groups (Therneau and Grambsch 2001). This allowed inclusion of right‐censored observations (i.e., plants that did not flower before the end of the experiment, in F1s *n* = 3). The Kaplan–Meier survival analysis estimates the probability a plant has not yet flowered, and allows comparisons between groups (e.g., priority × aging treatment). The same analysis was used to evaluate differences in the distributions of flowering onset between aging treatment × provisioning priority groups in the F2 generation (right censored *n* = 20).

A drawback of the Kaplan–Meier approach is that it cannot accommodate random effects terms, and thus we could not control for maternal identity and block (i.e., position in the greenhouse). Consequently, we also built linear mixed effects models (Bates et al. 2015) with censored observations excluded to account for the influence of these random effect terms (Tables S1, S3). We validated assumptions of variance, independence of errors, and normal distribution of residuals.

We used flowering time data from the F2 generation, which was obtained by a balanced experimental design and free of a plastic storage effect, to run simulations that estimated the expected invisible fraction bias arising from underrepresentation of last‐produced seed in the ancestral population. We sampled flowering time data from F2 control plants unequally, for each priority and aging treatment group, at proportions observed from F1 survivorship. As is the reality for most resurrection experiments, we calculated the mean trait value (i.e., first flowering day) per aging treatment group, as if seed priority was unknown. Flowering day bias is the difference in mean first flowering day between control (i.e., descendant) and aged (i.e., ancestral) groups. Each resample and bias calculation was repeated 10,000 times to understand the extent of an invisible fraction bias operating at the intermediate mortality level (26%) that we induced.

We also sought to understand how strong the priority effect (i.e., differential survivorship) must be to introduce substantial bias in the ancestral flowering day estimate. In a second simulation, aged and control groups have equal sample sizes to control for mortality effects independent of the priority effect. However, for the aged cohort, the ratio of first vs. last produced individuals ranges from 50:50 to 95:5 (by intervals of 5). We run 10,000 iterations for each priority effect strength (i.e., ratio).

All statistical analyses and figures were produced using R version 4.4.1 (R Core Team 2024).

## 3 Results

In the F0 generation, the early flowering (EE) plants flowered approximately two weeks earlier than the late flowering plants (LL), confirming plants form two distinct genetic lines (Figure S1). The number of seed pods produced by a plant was significantly influenced by the number of flowers produced and genetic line, but not block, suggesting there were no unintentional fitness differences that arose between blocks (Figure S2, F = 77.43, df = 60, *p* < 0.001, adjusted *R* ^2^ = 0.78).

### 3.1 Survivorship Differences After Aging

**FIGURE 3:**
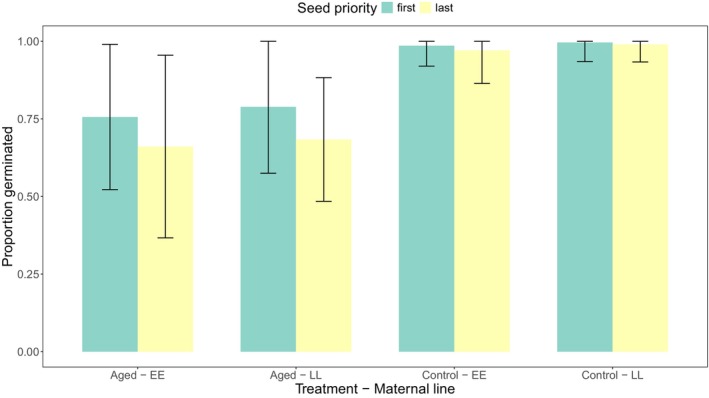
Germination success in the F1 generation based on seed priority, maternal line, and treatment (*n* = 3626; 3–5 per priority group × maternal line × aging treatment). Green indicates seeds produced first on a maternal plant (“first”) and yellow indicates seeds produced last on a maternal plant (“last”). Maternal line is indicated by EE (early flowering line) or LL (late flowering line). Treatment refers to rapid aging of F1 seeds to mimic storage conditions, indicated as aged or control. Error bars show standard deviation.

In the F1 refresher generation, seeds subject to a rapid physiological aging treatment had 26% lower germination versus control seeds (Figure 3, Table 1). Germination rates did not differ significantly with respect to seed priority, maternal line, maternal block, or an interaction between aging treatment and seed priority (Table 1). Last produced seeds germinated approximately 10% less than first produced seeds when aged; however, the non‐significant interaction term suggests the proposed bias‐generating mechanism did not occur outside of chance alone. Additionally, high germination in F1 control groups (99% on average) indicates that germination differences largely reflect mortality, rather than dormancy (Figure 3). Seed dormancy rates are highest in *Brassica* closer to harvesting time (Haile and Shirtliffe 2014); here F1 seeds were planted < 1 month after harvesting. Germination rates returned to high levels (on average 98%) in the F2 generation (Figure S5).

**TABLE 1:** Main and interaction effects of treatment, seed priority, block, and maternal line on germination success in the F1 generation using a binomial GLMM with a logit link term.

Term	Estimate	Standard error	*z*‐value	*p*
Intercept	4.39	0.55	7.97	**< 2e‐14**
Treatment (aged)	−3.39	0.42	−8.13	**4.44e‐16**
Seed priority (last)	−0.72	0.50	−1.43	0.15
Block (2)	0.21	0.15	1.35	0.18
Maternal line (LL)	−0.07	0.31	−0.23	0.82
Treatment: Seed priority	0.14	0.51	0.27	0.79

*Note:* Statistically significant *p*‐values (*p* < 0.05) are bolded.

### 3.2 Flowering Time Differences After Aging

**FIGURE 4:**
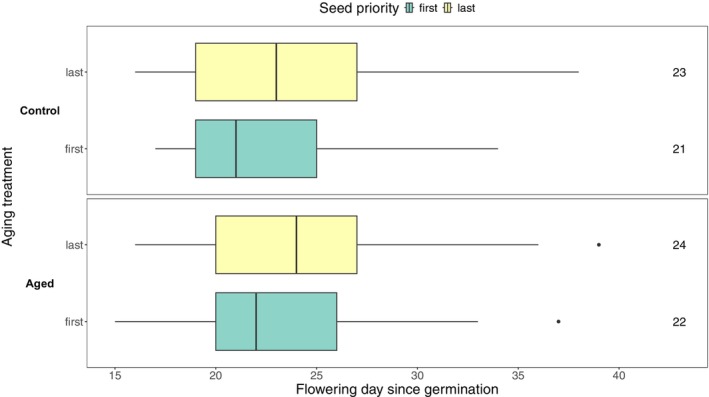
First flowering day relative to germination day for the F1 generation based on seed priority and aging treatment (*n* = 695). Numbers on the right indicate median first flowering day. Note that censored observations (i.e., plants that did not flower before the end of the experiment, *n* = 3) were excluded. The upper panel shows plants reared from control seeds, and the lower panel shows plants from rapidly aged seeds. Seed priority denotes seeds produced first on a maternal plant (“first” in green) and last on a maternal plant (“last” in yellow). See Figure S3 for F1 flowering time curves.

There were no detectable flowering time differences between the aged and control cohorts in the F1 generation (Table 2, Figure 4). Aged individuals flowered approximately one day later than controls; however, pairwise comparisons from both the Kaplan–Meier analysis and linear mixed effects model (which additionally accounted for maternal identity and position in the greenhouse) revealed no significant differences between any groups (Figure 4, Tables S1, S2). Thus, flowering time did not exhibit a plastic response to the aging treatment.

**TABLE 2:** *P*‐values from pairwise comparisons of Kaplan–Meier flowering day curves for each control vs. aged seed priority cohort in the F1 generation.

Pairwise comparison	*p*
Control–first vs. Aged–first	0.08
Control–last vs. Aged–last	0.19

**FIGURE 5:**
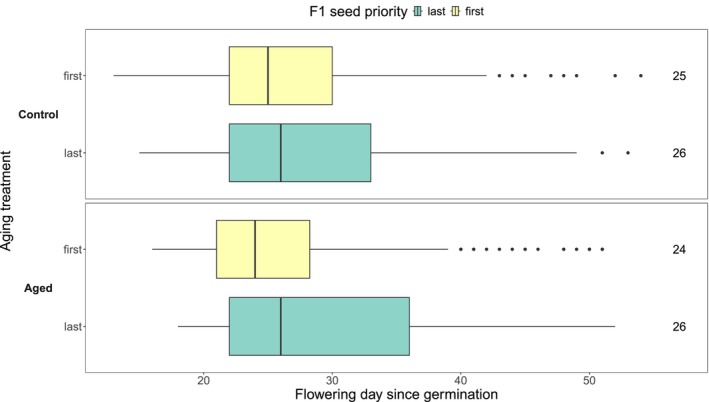
First flowering day relative to germination day in the F2 generation based on F1 seed priority and aging treatment (*n* = 941). Numbers on the right indicate median first flowering day. Note that censored observations (i.e., plants that did not flower before the end of the experiment, *n* = 20) were excluded. The upper panel shows plants reared from control seeds, and the lower panel shows plants from rapidly aged seeds. Yellow indicates offspring of seeds produced first on a maternal plant (“first”) and green indicates offspring of seeds produced last on a maternal plant (“last”). See Figure S4 for F2 flowering time curves.

In the F2 generation, neither the Kaplan–Meier test (Figure S4, Table 3) nor linear mixed effects model (Tables S3, S4) detected significant pairwise differences in flowering time with aging treatment. This result was expected, because the balanced sampling design should have removed any invisible fraction effect, and the F1 served as a “refresher” generation that removed any plastic response to the aging treatment. The F2 generation also did not exhibit effects of priority group on flowering time (Table 3, Figure S4, Table S4). Individuals derived from aged, last priority seed flowered, on average, approximately two days later than those from aged, first priority (Figure 5). The flowering time difference between first‐and last‐priority seeds in aged seed is consistent with the last‐priority seeds carrying more alleles for late flowering, as hypothesized in Figure 1. However, the absence of a flowering time difference between first and last priority seeds in the control treatment was unexpected.

**TABLE 3:** *P*‐values from pairwise comparisons of Kaplan–Meier flowering day curves for each control vs. aged seed priority cohort in the F2 generation.

Pairwise comparison	*p*
Control–first vs. Aged–first	0.36
Control–last vs. Aged–last	0.14

### 3.3 Estimating an Invisible Fraction Bias

Equal sampling of each aging treatment × priority group was required to ensure consistent power to estimate flowering time means, but by eliminating inequalities in survivorship, we masked any invisible fraction effects. We therefore used the observed germination (from F1) and flowering time data (from F2) to parameterize two simulations quantifying the potential for an invisible fraction bias. The first showed that the mortality level observed in the greenhouse experiment (26%) resulted in no bias on average (mean difference between aged and control = 0.04 days, *n* = 10,000 resamples). Bias from individual iterations ranged from −3.61 to 3.21 days.

**FIGURE 6:**
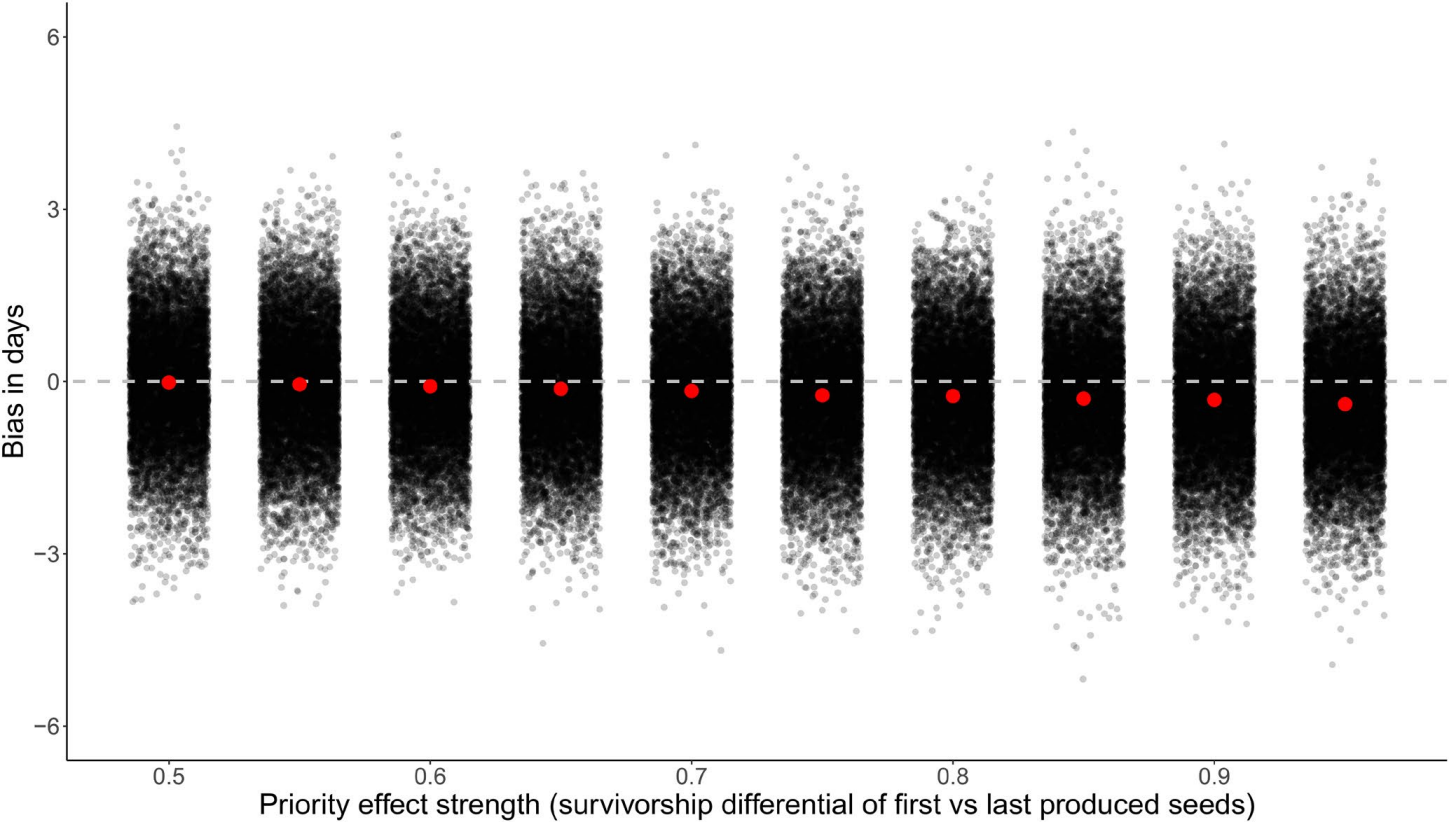
The invisible fraction bias on F2 first flowering day. Bias is calculated at various survivorship asymmetries between first and last produced seeds, from perfectly equal survival (0.5) to a priority effect that strongly favors survival of F1 first produced seeds (0.95). The dashed gray line indicates zero bias and red dots represent mean values.

The second simulation sampled from observed data, varying the proportion of seeds drawn from the two priority groups, asked how strong the effect of priority on seed survival must be to induce a non‐trivial invisible fraction bias. When the two priority groups are equally represented in the sample, no data are missing, and so the bias is essentially zero. Bias remains small as representation of the priority group increases relative to the last, i.e., when late birth order decreases seed longevity (Figure 6). At the extreme, when last priority individuals make up only 5% of the sample, the estimated mean flowering time is less than a day earlier than the unbiased mean. Even when the missing fraction is large, the resulting bias is small.

## 4 Discussion

The resurrection approach is a powerful tool for assessing adaptive evolutionary response to global change (Franks et al. 2018). When archived ancestral propagules from a population are available, they can be revived and grown side‐by‐side with propagules collected from the contemporary, descendant generation. Since they grow in the same environment, phenotypic differences between ancestors and descendants signify genetic (evolutionary) differences. We explored two biases that can distort the estimate of the ancestral mean phenotype, which is the baseline for estimating evolutionary response. The first is the storage effect, which is a plastic response of post‐emergence phenotypes to the environmental stress experienced over the storage period. The second effect is the invisible fraction bias, which can arise when mortality during storage is non‐random (Franks et al. 2018; Weis 2018). If storage survival traits are genetically correlated to the post‐emergence traits of interest, the surviving individuals will express phenotypes sampled non‐randomly from the phenotypic distribution that would arise under full survival. Either or both of these biases can lead to over or underestimation of the evolutionary response in a resurrection study. Thus, understanding the potential magnitude of these biases is essential to reliably interpret results.

Using lines of *B. rapa* we tested whether provisioning priority (i.e., birth order of propagules on a plant) influenced seed survival in storage. The survivors were grown to determine if their flowering times varied with birth order. These data were used in simulations exploring the potential magnitude of the invisible fraction bias on the flowering time mean as a function of provisioning priority effect on seed survival.

Our results showed that any invisible fraction bias arising through the priority mechanism is undetectably small. This is good news. This study was designed to maximize the potential for such a bias, and so the negative result argues that previously published resurrection studies showing rapid evolution of flowering time were unlikely skewed by an invisible fraction effect. However, a storage effect bias was evidenced by the small difference between the aged and control groups found in the F1 generation, which disappeared in the F2. Similarly, Franks et al. (2019) found that most of the phenotypic differences between control and rapidly aged plants disappeared in the F2 generation. Together, these results argue for the importance of running resurrected propagules through a refresher generation, so that ancestor–descendant differences are not distorted by plastic responses to the storage environment.

Our proposed mechanism for a bias estimate of flowering time in the ancestral generation rested on two conditions: (1) late flowering alleles should be more frequent in the last seeds produced on plants (compare the frequency of L allele in Figure 1C,E vs. Figure 1D,F), and (2) the last seeds produced by plants are less likely to survive storage than are the first seeds (compare proportion of seeds dying in Figure 1C,E vs. Figure 1D,F). The absence of detected bias in our study arises because, at least in *B. rapa* , there was only weak support for condition 1, and we detected the predicted difference in survivorship but lacked statistical support for condition 2. We discuss each condition in turn below.

Considering condition 1, if late flowering alleles were more commonly included in the last‐priority seeds, then we would expect, for example, the control–last priority seeds from EE plants to have a later flowering onset than the first priority seeds within this same treatment and maternal line, but the distributions are nearly the same (Figure S6). Similarly, we would expect priority seeds on late flowering plants to have an earlier flowering distribution than last priority seeds, but the observed difference is weak (Figure S6), relative to the 14‐day effect of late alleles in the F0 generation (Figure S1). Two non‐exclusive factors could be contributing. First, flowering time distributions in the F1 suggest a genetic dominance of early flowering alleles, so even though late flowering alleles are more represented in the last produced flowers, their effect is not evident. In alignment with the dominance hypothesis, Bonner et al. (2019: Figure 3B) found that the mean flowering time of hybrid progeny of these same selection lines is closer to that of the early line than of the late line. Second, even though we used lines intended to maximize the opportunity to detect an invisible fraction effect, there may have been insufficient overlap in the flowering schedules between the two flowering lines. For example, when the last produced flowers from EE plants were open between counts 3 and 4, most potential pollen donors were likewise from the early line (Figure 2C). Similarly, between counts 4 and 5, when we collected first‐produced seeds from late‐line plants, most pollen donors contributed late flowering alleles. The strong assortative mating induced by the experimental setup may have prevented production of sufficient EL offspring to produce a strong bias (Figure 1).

Regarding condition 2, although aged, last produced seeds germinated 10% less than aged, first produced, there was no statistical support for differences in seed survivorship between priority groups (Table 1, Figure 3). One might assume that first produced seeds that are prioritized by maternal provisioning have greater mass than later produced siblings, but seed mass was remarkably consistent between priority groups (Figure S7). Franks et al. (2019) demonstrated that, in fact, heavier seeds showed lower germination success following aging in rapid‐cycling *B. rapa* , although this phenomenon is not consistent across taxa (e.g., Genna et al. 2020). It is therefore unlikely that the weak survivorship differences between priority groups for aged F1 individuals were driven by resource quantity (Figure 3). We did not explore whether mass loss after aging differs with provisioning priority, but this is a potential future line of inquiry.

Small differences in germination between ancestors and descendants theoretically preclude a strong invisible fraction bias (Weis 2018), but the difference at which this bias begins operating, and has influenced past resurrection studies, has been unclear. The invisible fraction has yet only been recorded under very strong selective pressure (69% germination failure) on the ancestral cohort (Franks et al. 2019). Our results suggest resurrection experiments with moderate failure rates (26%) of ancestral propagules should be reliable. Less than half (24/55 = 43.6%) of plant resurrection studies reported germination—of these, germination rate differed on average 7.3% (ranging from −80% to 88%) between ancestral and descendent cohorts, suggesting that an invisible fraction bias is unlikely to be affecting the literature on average, although some studies may be compromised when germination is highly asymmetrical between generations. Use of a refresher generation by most studies (69.1%) evaded introduction of a plastic stress response to long‐term storage. Failure to control for the storage condition bias can also obscure estimates of the true ancestral trait baseline, probably more than selection in storage (Franks et al. 2019). Consequently, we recommend two best practices when executing resurrection experiments. First, using a refresher generation is critical to generate results free of a plastic response to storage stress. Second, reporting germination rate, ideally on a per seed basis, quantifies the selective sieve imposed on the ancestral generation. For some taxa, breaking seed dormancy may be necessary (e.g., Mousavi et al. 2011), or else dormant propagules will also contribute to the unmeasured invisible fraction, whenever germination probability correlates to the trait of interest.

In this study system, the priority effect introduced little bias in estimating flowering time, indicating that documenting seed order is likely not important for seedbank managers. When executing the resurrection approach in diverse systems, knowing the underlying genetic architecture determining a correlation between traits conferring propagule longevity and the adult focal trait is the ideal approach to estimate an invisible fraction problem. A robust understanding of the genetic underpinnings of non‐random propagule loss might be prioritized for a handful of commonly used model taxa, such as those stored by Project Baseline (Etterson et al. 2016).

## Author Contributions

**Sarah M. Ravoth:** conceptualization (equal), formal analysis (lead), investigation (equal), methodology (equal), project administration (equal), visualization (lead), writing – original draft (lead), writing – review and editing (lead). **Ariane Mooney:** investigation (equal), methodology (supporting), project administration (equal), writing – review and editing (supporting). **Emily J. Austen:** conceptualization (supporting), supervision (supporting), writing – review and editing (equal). **Alice DesRoches:** investigation (supporting), writing – review and editing (supporting). **A. J. Westerhof:** investigation (supporting), writing – review and editing (supporting). **Arthur E. Weis:** conceptualization (equal), methodology (equal), project administration (equal), supervision (lead), writing – original draft (supporting), writing – review and editing (equal).

## Conflicts of Interest

The authors declare no conflicts of interest.

## Supporting information


**Data S1:** ece373101‐sup‐0001‐Supinfo.docx.

## Data Availability

Data and scripts are available on Figshare at https://doi.org/10.6084/m9.figshare.30032077.
