# Supplementary material for: Investigating a Potential Bias in Resurrection Experiments to Measure Adaptive Evolution of Flowering Time
Source: Ecol Evol. 2026 Mar 23;16(3):e73101. doi: 10.1002/ece3.73101 (PMC13093673; doi:10.1002/ece3.73101)
Supplement: Supplementary file 1 — Data S1: ece373101‐sup‐0001‐Supinfo.docx. [file ECE3-16-e73101-s001.docx]

**Appendix S1: Supplementary tables & figures**


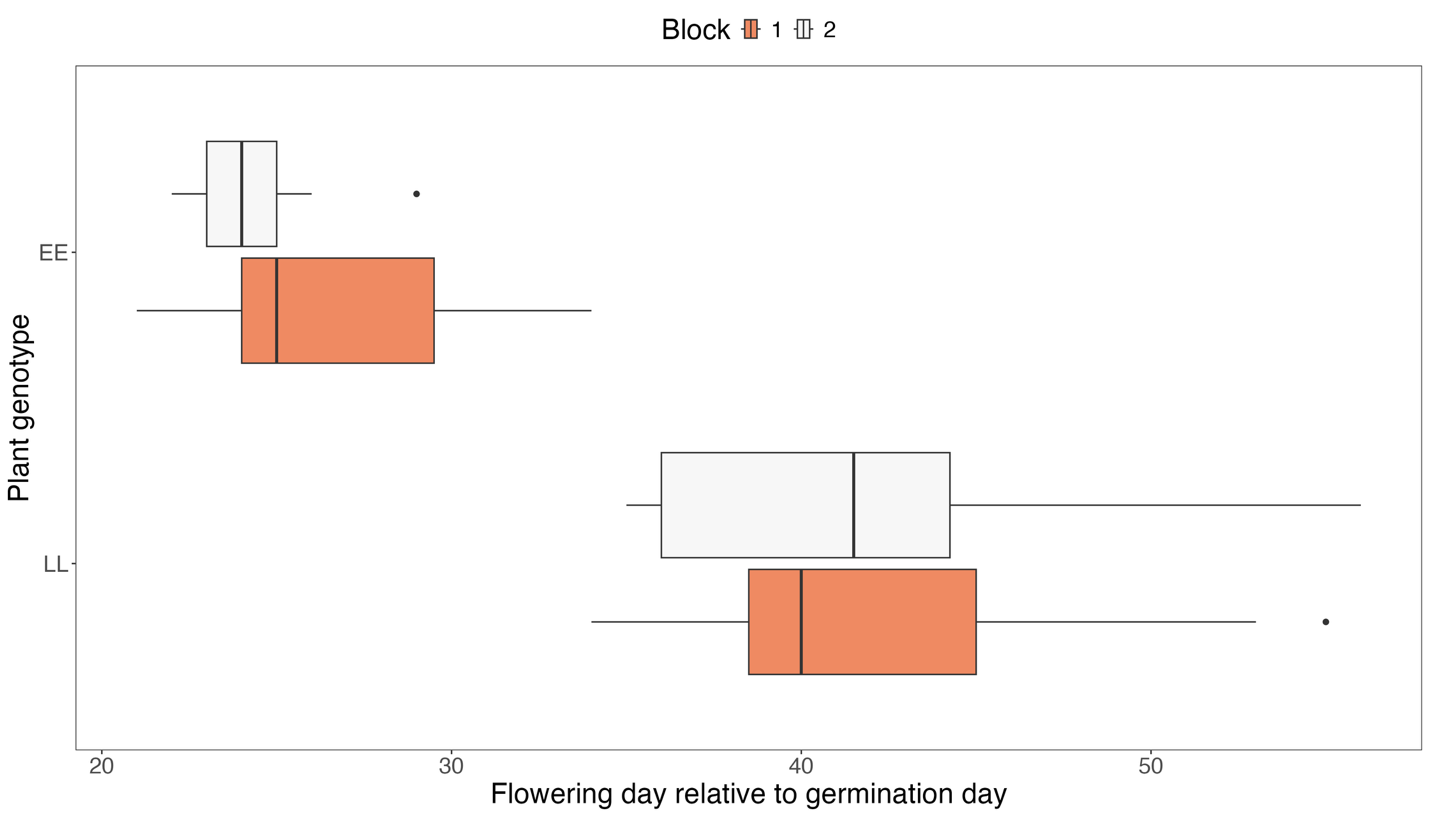


Figure S1. First flowering day relative in the F0 generation. Colour indicates the block plants were growing in. First flowering day on the x-axis is relative to each plants’ germination day. Plants from the early flowering line (“EE”) flowered on average two weeks earlier than plants from the late flowering line (“LL”) in both blocks.


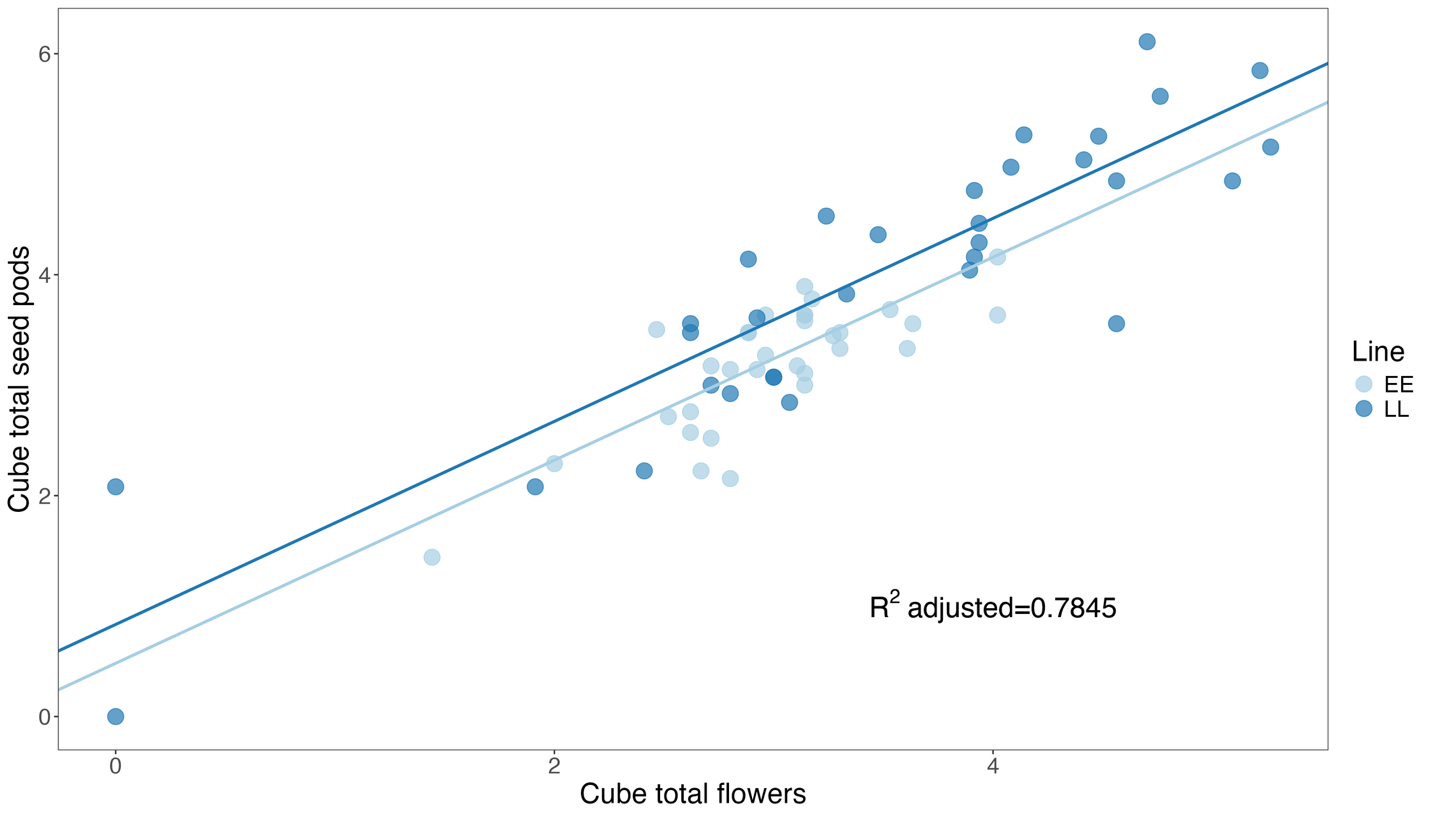


Figure S2. Flowers versus seed pod counts produced by F0 plants from blocks 1 and 2. Light blue indicates individuals from the early flowering line (“EE”) and dark blue indicates individuals from the late flowering line (“LL”). The relationship between flower and seed pod production is congruent between lines, except that LL plants tend to produce more flowers and seed pods than EE plants. A linear model with flower count, genetic line, and block as fixed effects well explained variation in seed pod count (R^2^ adjusted=0.7845).

Figure S3. First flowering day curves in the F1 generation, grouped by seed priority. The x-axis shows days since germination, and the y-axis indicates the probability a plant has not yet flowered. Small crosses on the flowering curves indicate a right-censored observation (i.e., a plant that did not flower before the end of the experiment, n=3). Line colour indicates control vs aged treatment and shading indicates the 95% confidence interval. This figure corresponds to Figure 4 F1 flowering time boxplots.

Figure S4. First flowering day curves in the F2 generation, grouped by F1 seed priority. The x-axis shows days since germination, and the y-axis indicates the probability a plant has not yet flowered. Small crosses on the flowering curves indicate a right-censored observation (i.e., a plant that did not flower before the end of the experiment, n=20). Line colour indicates F1 control vs aged treatment and the shading indicates the 95% confidence interval. This figure corresponds to Figure 5 F2 flowering time boxplots.


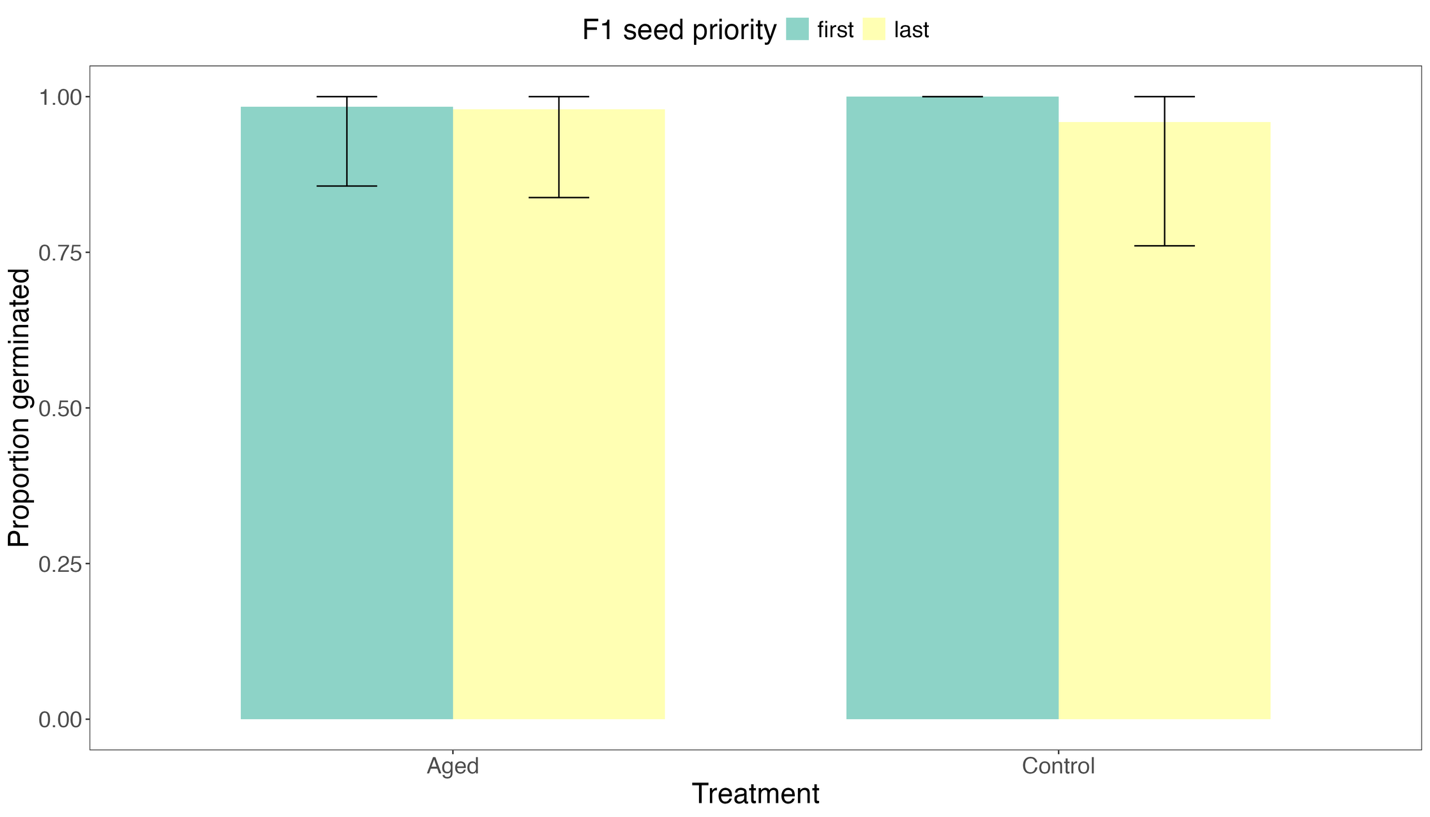


Figure S5. Germination success in the F2 generation based on seed priority and aging treatment (n=980). Green indicates seeds produced first on a maternal plant (“first”) and yellow indicates seeds produced last on a maternal plant (“last”). Treatment from the preceding F1 generation is indicated as aged or control. First produced control plants lack an error bar because there was no variation in germination.


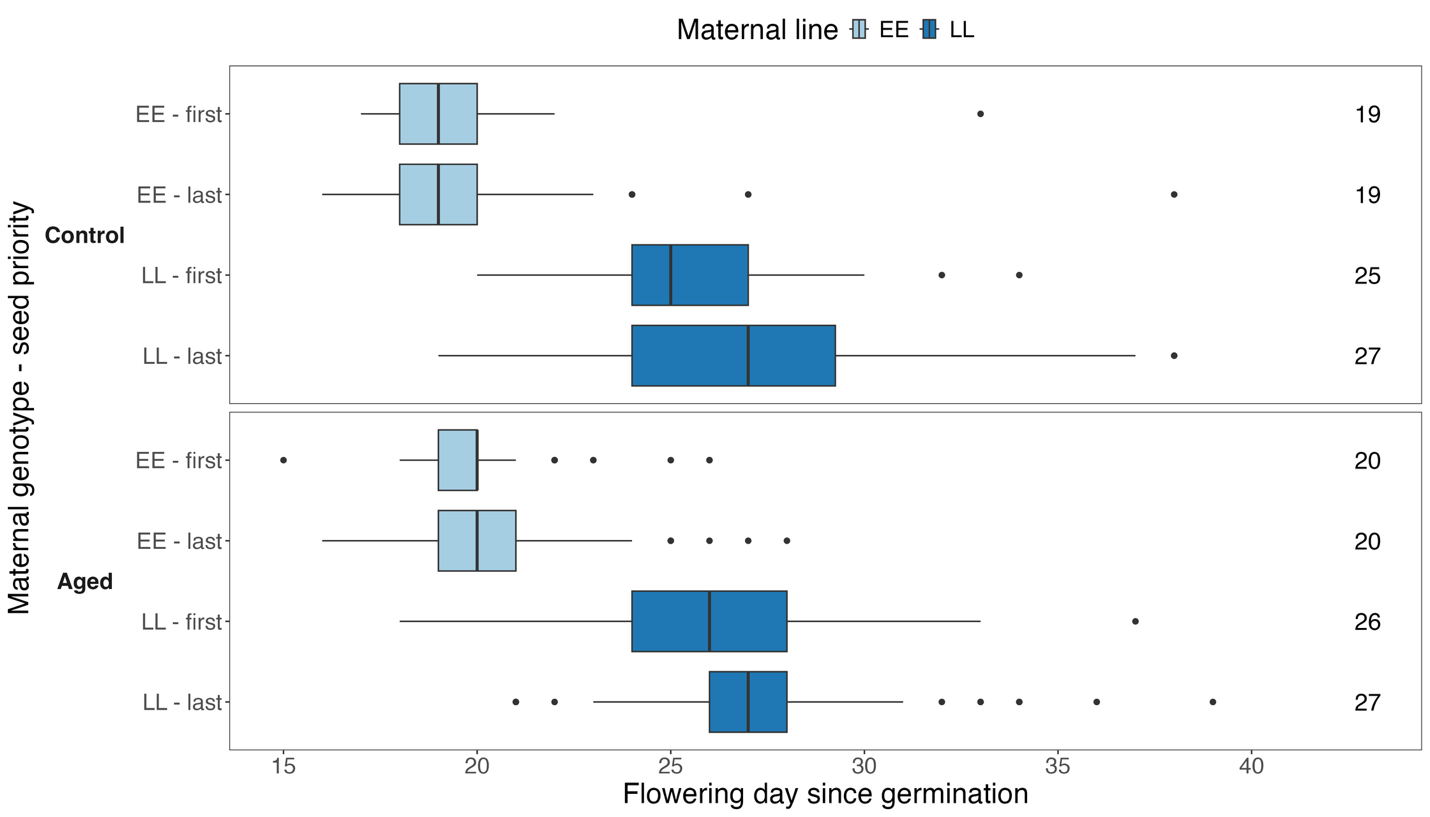


Figure S6. First flowering day relative to germination day in the F1 generation based on maternal line, seed priority, and ageing treatment (n=941). Numbers on the right indicate median first flowering day. Note that censored observations (i.e., plants that did not flower before the end of the experiment, n=20) were excluded. The upper panel shows plants reared from control seeds and the lower panel plants from rapidly aged seeds. Yellow indicates offspring of seeds produced first on a maternal plant (“first”) and green indicates offspring of seeds produced last on a maternal plant (“last”).


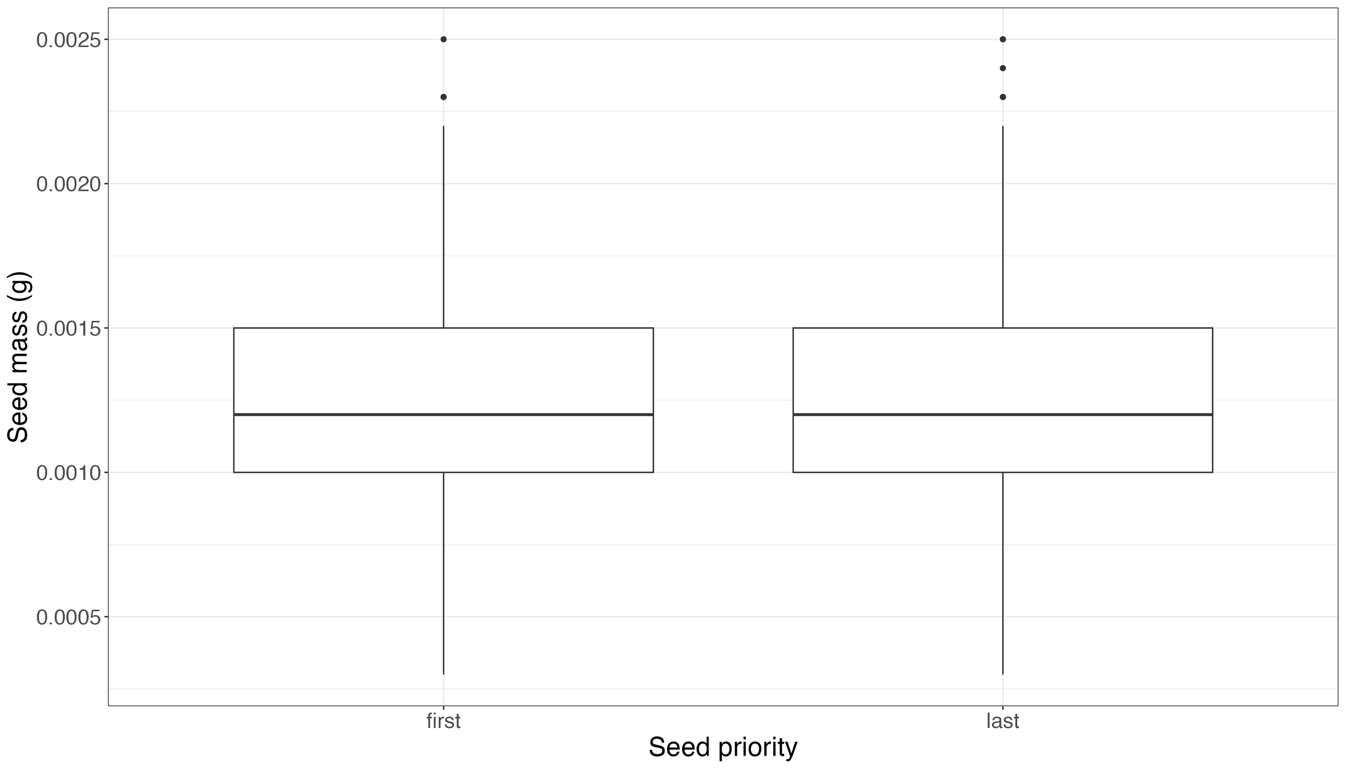


Figure S7. Seed mass (grams) of first and last produced seeds on a plant. Seed mass was weighed prior to aging.

Table S1. Main and interaction effects of treatment, seed priority, and their interaction for a linear mixed effects model of first flowering day in the F1 generation. Statistically significant p-values (p<0.05) are bolded. Respectively, random effects of maternal ID and planting flat accounted for 2.0% and 55.4% of random variance.

| *Term* | *Estimate* | *Standard error* | *t-value* | *p-value* |
| --- | --- | --- | --- | --- |
| Intercept | 22.16 | 1.50 | 14.77 | **2e-16** |
| Treatment (aged) | 0.75 | 2.12 | 0.35 | 0.40 |
| Seed priority (last) | 0.98 | 2.12 | 0.46 | 0.50 |
| Treatment:Seed priority | -2.93 | 2.67 | -1.10 | 0.27 |

Table S2. Pairwise comparisons of flowering time between aged vs. control groups for each seed priority pair in the F1 generation from a linear mixed effects model. Contrasts were planned and corrections for multiple comparisons were not applied. Statistically significant p-values (p<0.05) are bolded.

| *Contrast* | *Estimate* | *Upper CL* | *Lower CL* | *Standard error* | *Degrees of freedom* | *t-ratio* | *p-value* |
| --- | --- | --- | --- | --- | --- | --- | --- |
| Control–first vs. Aged–first | -0.75 | 3.63 | -5.13 | 2.12 | 23.3 | -0.35 | 0.73 |
| Control–last vs. Aged–last | 2.17 | 5.51 | -1.16 | 1.63 | 26.5 | 1.34 | 0.19 |

Table S3. Main and interaction effects of treatment, seed priority, and their interaction for a linear mixed effects model of first flowering day in the F2 generation. Statistically significant p-values (p<0.05) are bolded. Greenhouse position accounted 1.5% of random variance.

| *Term* | *Estimate* | *Standard error* | *Degrees of freedom* | *t-value* | *p-value* |
| --- | --- | --- | --- | --- | --- |
| Intercept | 26.87 | 0.56 | 125.84 | 48.38 | **<2e-16** |
| Treatment (aged) | -0.66 | 0.73 | 926.33 | -0.91 | 0.36 |
| Seed priority (last) | 0.87 | 0.73 | 924.05 | 1.19 | 0.24 |
| Treatment:Seed priority | 1.82 | 1.04 | 927.01 | 1.76 | 0.08 |

Table S4. Pairwise comparisons of flowering time between aged vs. control groups for each seed priority group in the F2 generation from a linear mixed effects model. Contrasts were planned and corrections for multiple comparisons were not applied. Statistically significant p-values (p<0.05) are bolded.

| *Contrast* | *Estimate* | *Upper CL* | *Lower CL* | *Standard error* | *Degrees of freedom* | *t-ratio* | *p-value* |
| --- | --- | --- | --- | --- | --- | --- | --- |
| Control–first vs. Aged–first | 0.66 | -0.77 | 2.09 | 0.73 | 926 | 0.91 | 0.37 |
| Control–last vs. Aged–last | -1.16 | -2.62 | 0.29 | 0.74 | 928 | -1.57 | 0.12 |
